# Molecular Insights into the Adsorption of Deposit Control Additives from Hydrocarbon Fuels

**DOI:** 10.1021/acs.langmuir.4c04368

**Published:** 2025-01-16

**Authors:** Carlos Corral-Casas, Carlos Ayestarán Latorre, Chiara Gattinoni, Mark Brewer, Jörn Karl, Daniele Dini, James P. Ewen

**Affiliations:** † Department of Mechanical Engineering, Imperial College London, South Kensington Campus, London SW7 2BX, United Kingdom; ‡ Department of Physics, King’s College London, Strand Campus, London WC2R 2LS, United Kingdom; ¶ Shell Global Solutions International B.V., Grasweg 39, 1031 HW Amsterdam, The Netherlands; § Shell Global Solutions (Deutschland) GmbH, Hohe-Schaar-Straße 36, 21107 Hamburg, Germany

## Abstract

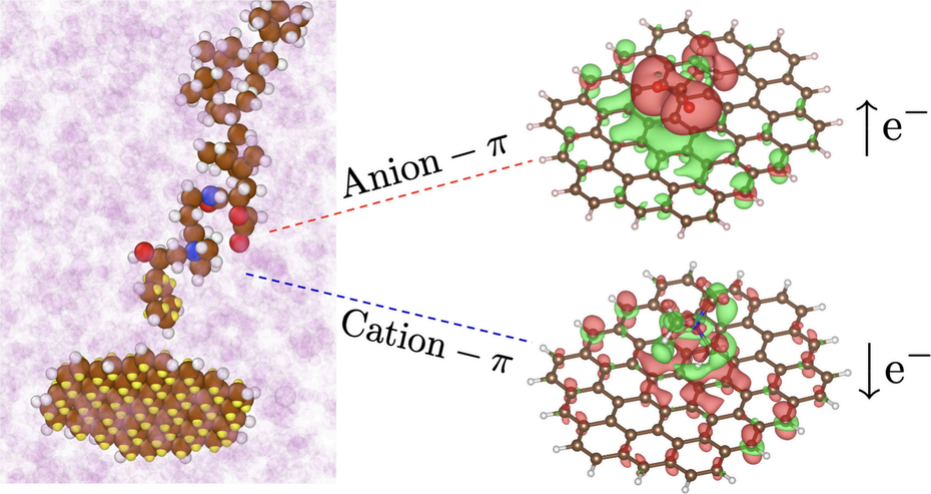

Engine deposits can reduce performance and increase emissions, particularly for modern direct-injection fuel delivery systems. Surfactants known as deposit control additives (DCAs) adsorb and self-assemble on the surface of deposit precursors to keep them suspended in the fuel. Here, we show how molecular simulations can be used to virtually screen the ability of surfactants to bind to polyaromatic hydrocarbons, comprising a major class of carbonaceous deposits. We use molecular dynamics with the adaptive biasing force method to generate the potential of mean force as a function of the vertical distance between the surfactants and deposits in gasoline and diesel fuel surrogates. We find that a zwitterionic surfactant outperforms a conventional polyisobutylene succinimide for binding to these aromatic species. The amine groups in the succinimide headgroup only weakly adsorb on the polyaromatic deposit, while additional functional groups in the zwitterionic surfactant, particularly the quarternary ammonium ion, markedly enhance the binding strength. We decompose the adsorption free energies of the surfactants into their entropic and enthalpic components, to find that the latter dominates the attraction from these non-aqueous solvents. The adsorption free energy of both surfactants is slightly weaker from *n*-hexadecane (diesel) than iso-octane (gasoline), which is due to the larger steric barrier from stronger molecular layering of the former on the deposit. Density functional theory calculations of the adsorption of DCA fragments validate the force field used in the molecular dynamics simulations and provide further insights into the nature of the intermolecular interactions. The approach introduced here shows considerable promise for accelerating the discovery of novel DCAs to facilitate more advanced fuel formulations to reduce emissions.

## Introduction

Transportation is currently the sector with the highest reliance on fossil fuels and accounts for more than a third of global CO_2_ emissions.^1^ Despite increasing electrification of the global transport fleet, the vast majority of vehicles still use fossil fuel-powered internal combustion engines (ICEs).^2^ It is therefore crucial to increase the efficiency of ICEs to ensure that fuel consumption and the subsequent CO_2_ emissions are minimized.^3^ A major issue affecting the efficiency of modern ICEs are deposits that form on metal surfaces inside the engine, particularly those on the fuel injectors.^4^ These deposits have long been recognized as a problem in diesel-powered compression ignition ICEs, where recent attention has focused on internal injector deposits.^5^ Historically, injector deposits have been less of an issue for gasoline-powered spark ignition ICEs with port fuel injection.^4^ The majority of new spark ignition engines now use gasoline direct ignition technology, which can deliver higher power output and better fuel economy than the port fuel technology, but at the expense of increased particulate matter emissions.^6^ Another related problem with the widespread adoption of gasoline direct ignition technology is that it has led to an increase in deposit-related issues for gasoline-powered ICEs.^4^

Deposits can lead to a decrease in power and fuel economy, as well as an increase in CO_2_ and pollutant emissions from ICEs.^7−9^ Recent studies have confirmed that polyaromatic hydrocarbons (PAHs) are major components of engine deposits formed in diesel and gasoline direct-injection engines.^10,11^ PAHs are formed from the breakdown of fuel molecules through thermo-oxidation, which is followed by polymerization of the degradation products.^12,13^ The PAH molecules, acting as deposit precursors, grow up to ∼800 Da^10^ and agglomerate in the fuel until they become insoluble and are deposited onto metal surfaces.^14^ Likewise, the degradation processes can be accelerated by these surfaces within the engine.^15^ It is worth noting that the size of the PAH molecules is similar to those found in soot (239–838 Da).^16^ Deposits contain a significant amount of oxygen when initially formed, which decreases with time while their aromatic content and porosity increases.^17^

The main mitigation strategy for engine deposits is to add surfactants to gasoline^18^ and diesel^19^ fuel formulations, which are known as detergents or deposit control additives (DCAs).^20^ DCAs act to prevent the agglomeration of PAHs to form insoluble deposits (keep-clean mode) and remove those that have previously formed on the metal surface (clean-up mode).^18,19^ The effectiveness of DCAs depends on their ability to adsorb onto deposits and metal surfaces via their polar headgroup. The nonpolar tail-group provides solubility in the affine fuel chemistry, besides preventing the deposits aggregation and further deposition through steric repulsion effects. The predominant and most effective chemistry for ICEs detergency over the past few decades has been based on polyisobutylene succinimide (PIBSI) additives.^21^ PIBSI derivatives are also used as dispersant additives in engine lubricants to stabilize soot or other deposit-related particles.^22,23^ PIBSIs have proven to be effective to neutralize acidic fuel degradation products (such as hydroperoxides) given the basic nature of secondary amines,^24^ although many different polyamine head-groups have been proposed in the literature.^25−31^ Other DCA chemistries have also been employed, such as alkylpropoxylates and alkylbutoxylates,^32^ polyhydroxystearic acid,^33^ polyether amines,^34^ Mannich bases,^34,35^ and quaternary ammonium salts.^36,37^

Experimental studies have measured adsorption isotherms for DCAs on model deposits from several nonpolar fluids. Gasoline, diesel, and lubricant surrogates, such as iso-octane,^28,32,38^ xylene,^25−27,33^ decalin,^33^ and *n*-dodecane^29−31,37^ have all been used as solvents. For reproducibility and cost reasons, these studies mostly used carbon black rather than real deposits, which is commonly employed as a surrogate for soot^39^ and engine deposits.^40^ Most of these studies observed adsorption isotherms that were consistent with Langmuir theory,^30,31,33,37^ which implies monolayer adsorption.^41^ Adsorption isotherms for some DCAs could not be fit with the standard Langmuir equation because a sharp increase in surface coverage was observed at high concentrations.^25,28,29,32,38^ This behavior has been attributed to hemimicelle^25,28^ or multilayer^29,32,38^ formation on the surfaces.

Molecular dynamics (MD) simulations have become an integral tool for the study of surfactant adsorption and self-assembly at solid–liquid interfaces for a wide range of industrially important systems.^42^ Prominent examples include formulated products such as paints,^43,44^ coatings,^45^ fuels,^46^ and lubricants.^47,48^ When compared to the nanosecond time scales accessible to conventional MD simulations, surfactant adsorption and self-assembly are relatively slow processes.^49^ The bottleneck of MD simulations lies in that there are high-energy barriers separating different metastable conformations, so that transitions between them are rare events^50^ and the system is said to be quasi nonergodic. In an attempt to extend MD to longer time scales, several enhanced sampling methods have been developed. Widely used techniques to improve the sampling efficiency within MD simulations rely on modifying the potential energy landscape by adding a bias potential to the Hamiltonian of the system.^51^ This approach underlies methods such as umbrella sampling^52^ and metadynamics.^53^ Methodologies framed on different theoretical principles have been devised too, such as the adaptive biasing force (ABF) technique^54,55^ that we adopt in this study. The ABF scheme involves the iterative modification of the average force felt by the system and, importantly, it does not require prior knowledge of the free energy landscape.^56^

Enhanced sampling methods have been applied to study surfactant adsorption at a wide range of aqueous interfaces, such as polymer–water,^44^ graphene–water,^57,58^ metal-water,^59^ metal oxide-water,^60^ mineral-water,^61,62^ and membrane-water.^63^ Far fewer studies have used enhanced sampling to investigate surfactant adsorption within non-aqueous solvents, and those that have been performed have focused on metal-glycol^64^ or metal oxide-alkane interfaces.^46−48^ We are not aware of any previous MD simulations with enhanced sampling of surfactant adsorption at PAH-alkane interfaces, which are of relevance to DCAs. In this study, we develop a computational framework suitable for the virtual screening of DCAs in terms of their propensity to bind to deposits. We use MD simulations with the ABF method to compare the adsorption of non-ionic PIBSI and a zwitterionic surfactant on PAHs from alkane solvents representative of gasoline and diesel fuels. To understand the thermodynamic driving force for adsorption in these systems, we decompose the free energy into the corresponding entropic and enthalpic contributions.^65^ Further insights are derived from density functional theory (DFT) calculations, which help to identify the relative importance of the different binding mechanisms as well as supporting MD results. We also check the PAH size dependency of the adsorption process and assess methodological considerations of relevance, such as the effect of an appropriate modeling of π-electrons on the adsorption free energy.

## Materials and Methods

### System Setup

**Figure 1:**
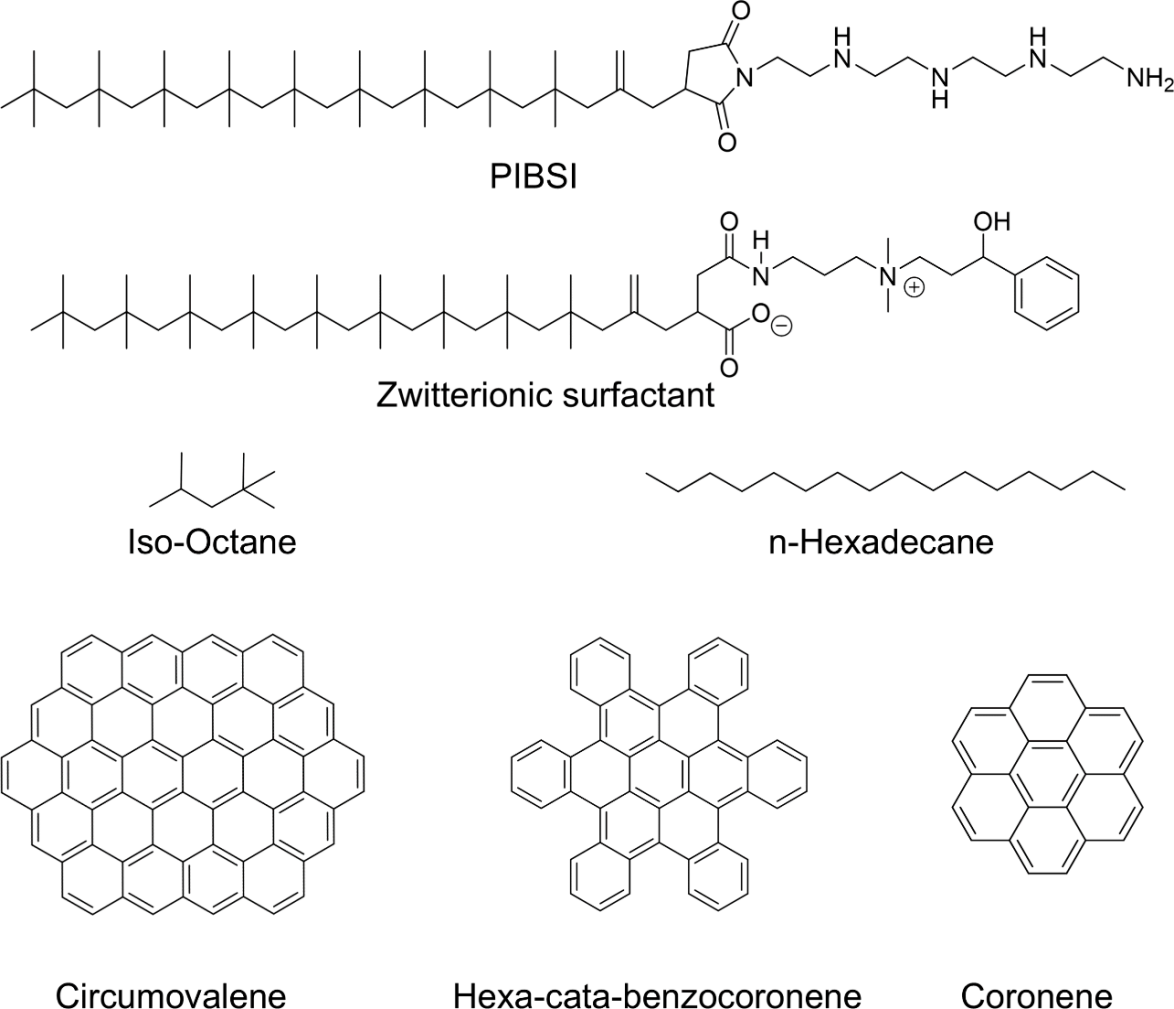
Chemical structures of the DCAs (PIBSI and zwitterionic surfactants), fuel surrogates (iso-octane and *n*-hexadecane), and PAH deposits (circumovalene, hexa-cata-benzocoronene, and coronene) considered in the MD simulations.

The molecular structures of the DCAs, fuels, and deposits used in the current study are shown in Figure 1. We compare two amphiphilic DCA chemistries with the same PIB tail-group consisting of ten repeat units of the isobutylene monomer. The first DCA is a well-known non-ionic surfactant based on the traditional PIBSI formulation. It contains a succinimide moiety acting as a hook group, bridging the aliphatic tail-group with tetraethylenepentamine (TEPA) acting as the headgroup. The second DCA is a zwitterionic surfactant with betaine chemistry,^66^ containing a cationic quaternary ammonium and an anionic carboxylate group. Previous experimental studies^37^ have highlighted the effectiveness of DCAs containing quaternary ammonium ions due to relatively strong cation-π interactions^67^ with the aromatic deposit. However, we are not aware of any studies accounting for a zwitterionic structure, comprising a counterionic functional group (such as the carboxylate anion) to constitute a neutral molecule. The zwitterionic surfactant also contains a phenyl group and an alcohol group, which will extend the range of possible binding modes through π–π and OH – π interactions,^68^ which is expected to lead to stronger adsorption.^31^ The molecular weight of the PIBSI and zwitterionic surfactants are 830 g mol^–1^ and 769 g mol^–1^, respectively, which is close to that found for commercial fuel DCAs.^5^ PIBSI is known to form inverse micelles in nonpolar solvents, with aggregation numbers ranging from 3 to 5.^24^ Likewise, ionic-functionalized PIB (similar to our zwitterionic surfactant) also forms inverse micelles, generally with larger aggregation numbers of 4–14 depending on the cation and anion combination.^37^ However, as in most previous free energy studies of surfactants,^48^ we only consider single-molecule adsorption to understand the pure thermodynamics of adsorption. Likewise, we focus on the case in which the deposit precursor is dissolved in the base fuel, where the deposit-DCA interactions are not affected by metallic surfaces.

For the hydrocarbon base solvent, we select iso-octane (2,2,4-trimethylpentane) as a model for gasoline and cetane (*n*-hexadecane) to represent diesel, which have been used in previous MD simulations as single-component gasoline^46^ and diesel^69^ fuel surrogates. The model deposits are PAHs with no heteroatoms, such as the circumovalene molecule  that we use for most of the simulations, which is the largest PAH molecule identified in the experiments.^10^ We also perform a subset of simulations with smaller PAH molecules coronene  and hexa-cata-benzocoronene  to probe deposit size effects on the adsorption process.

**Figure 2:**
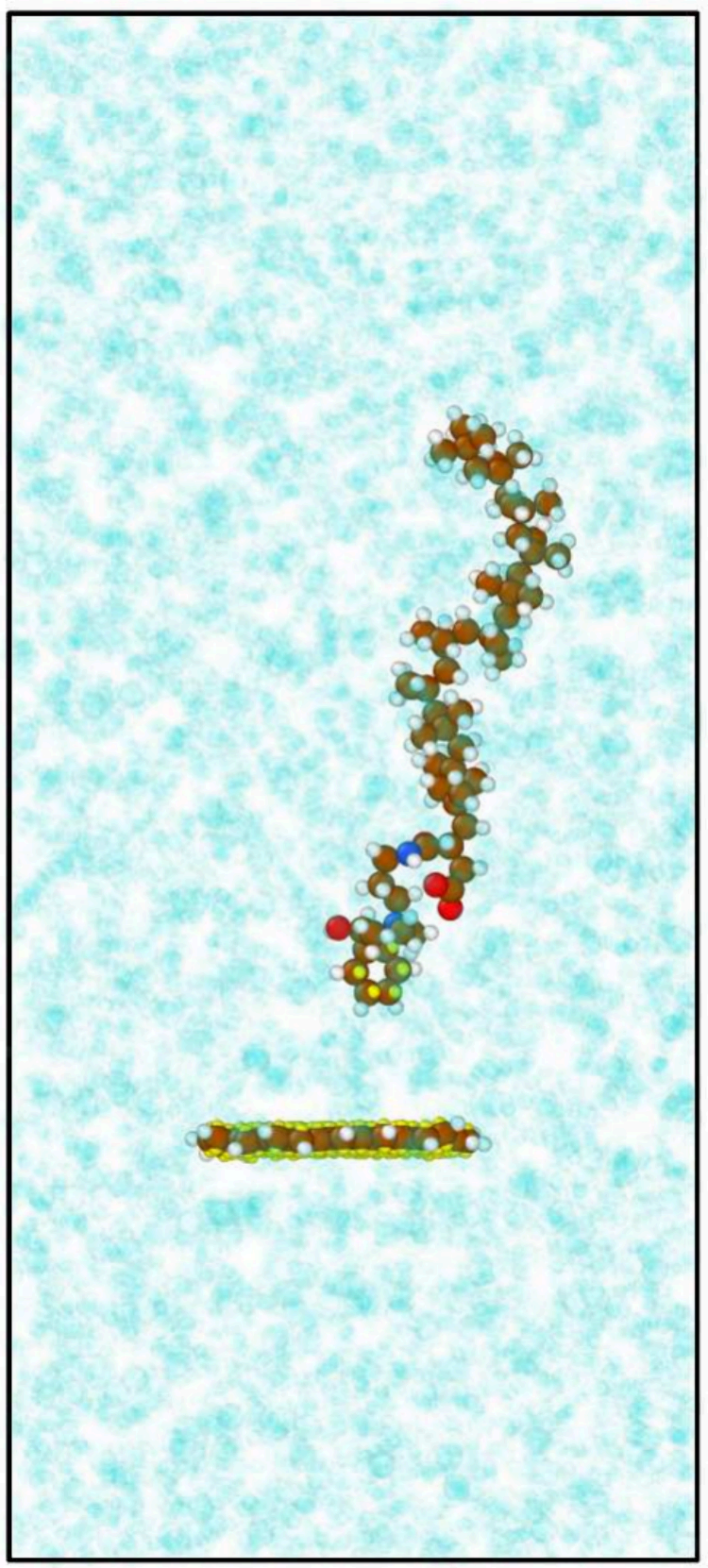
Snapshot of the system setup for the ABF-MD simulations. The deposit and the surfactant are shown using a ball-and-stick model, and the base solvent is represented with transparency. C atoms are colored brown, H atoms white, O atoms red, and N atoms blue. Virtual π-electrons are colored yellow. Rendered using OVITO.^70^

All of the systems are constructed by using the Materials and Processes Simulations (MAPS) platform from Scienomics SARL. A single circumovalene molecules is fixed in place by fixing the positions of three of its atoms so that it cannot rotate, and a single DCA molecule was placed above the carbonaceous deposit at the center of the simulation cell. Afterward, either 500 iso-octane or 300 *n*-hexadecane molecules are randomly distributed by using the Amorphous Builder plugin in MAPS, which roughly corresponds to the expected experimental liquid density for the simulation cell size. A schematic of the simulation box is shown in Figure 2.

The different elements of the system are modeled using the all-atom optimized potential for liquid simulations (OPLS-AA) force field.^71−73^ The long-chain parametrization^74^ is needed to overcome the well-documented overestimate of the liquid-gel transition temperature that occurs for long-chain alkanes, e.g. *n*-hexadecane. Another shortcoming of additive force fields based on point charges, such as OPLS-AA, is that they underestimate the strength of important intermolecular interactions for the current study. Notably, these force fields neglect the formation of ion-induced dipoles, which underpin the cation-π interactions^75^ between the zwitterionic surfactant and the deposit. We overcome this limitation through the explicit consideration of π-electrons with virtual particles using parameters from the INTERFACE force field (IFF).^76,77^ Following the IFF approach, the multipoles along the cloud of conjugated π – electrons are more accurately described, allowing one to capture the physical behavior surrounding aromatic groups. An additional advantage is that no further parametrization is needed for its integration with the rest of the OPLS-AA parameters.^78^

### MD Procedure

MD simulations are performed using the Large-scale atomic molecular massively parallel simulator (LAMMPS) software.^79^ The velocity-Verlet algorithm is used with a time step of 1 fs. Periodic boundary conditions are applied in all three Cartesian directions. The SHAKE algorithm is included to constrain the equilibrium length values for all covalent bonds including H atoms.^80^ The nonbonded interactions include both the Lennard-Jones potential with a 12 Å cutoff and long-range Coulombic interactions. Geometric mean mixing rules are used to represent the cross interactions between different atom types. The long-range electrostatic interactions are solved in reciprocal space using the particle–particle particle-mesh (PPPM) algorithm^81^ with a relative force accuracy of 10^–5^.

The systems are first energy minimized using the conjugate gradient approach. Next, we equilibrate at 350 K and 1 atm for 1 ns in the isothermal–isobaric (NPT) ensemble. We choose 350 K as the target temperature since this is representative of the conditions inside engines and is below the boiling point of both solvents. Production runs are carried out in the canonical NVT ensemble at 350 K for at least 100 ns. A subset of simulations was also performed at 300 K, 325 K, and 370 K to check for temperature effects on adsorption. The temperature and pressure are controlled with a Nosé–Hoover^82,83^ thermostat and barostat with a time relaxation constant of 0.1 and 1.0 ps, respectively. The ABF routine is implemented using the colvars plugin for LAMMPS;^84^ an example of a configuration file, needed for enabling enhanced sampling, is provided in the Supporting Information.

#### Calculating the Adsorption PMF

One fundamental measure of the interactions responsible for the DCA adsorption process is the potential of mean force (PMF), which describes the free energy change as a function of the distance between the DCA headgroup and the deposit. The adsorption free energy is taken as the global minimum value of the PMF, quantifying the affinity of the surfactant for a given surface with respect to staying in the bulk of the solvent.^57^ For a reliable PMF production, one must sample distributions favoring regions of phase space that would be infrequently visited owing to the force field interactions,^51^ e.g. using ABF. The concept of the collective variable (*colvar*), ξ, lies at the core of these methodologies.^84^ For the particular problem of adsorption, the most physically meaningful colvar choice is the *z* – projected (vertical) distance between the binding groups and the deposit. This is because lateral movements along the *x* – and *y* – directions do not significantly alter the free energy as long as the adsorbate remains within the binding sites, and so the Euclidean distance might constitute a suboptimal colvar. Besides accounting for the physical nature of the problem at stake, it is also important to choose the collective variable so that the biasing force is not heavily affected by stochastic noise. Therefore, ξ involves all of the nonfixed atoms in the deposit and all the atoms in the DCA headgroup, to cancel out the noisy force terms from the rapidly oscillating bonded interactions. In this way, the variance of the instantaneous force estimator^85^ is lowered, improving the ABF convergence.

The first enhanced sampling strategy consists on stratifying the relevant range of the collective variable, e.g. 0 ≤ ξ ≤ *r*_*cut*_, where *r*_*cut*_ is the cutoff distance from which the free energy no longer changes. By confining the region of phase space that can be examined to a small range of the colvar, a more comprehensive spectrum of conformations can be developed at the same computational expense. In this work, the stratification scheme is applied such that the range of collective variables is divided in windows that are 3 Å wide. The initial configurations for each window are obtained by using steered molecular dynamics,^86^ where the polar headgroup is pushed toward a prescribed distance with respect to the deposit. Standard harmonic potentials, which act only outside the colvar range, are applied to keep the system inside the windows^55^ during the equilibration and production runs. These restraints act like walls against which the groups related to the ξ definition will bounce. A force constant of at least 80 kcal mol^–1^ Å^–1^ is used to impede accumulation of samples near the borders, which would compromise the homogeneous inspection of phase space.^87^

The second strategy consists of using an importance sampling approach in order to sample all ξ values with an equal probability regardless of the ruggedness of the potential energy landscape. The ABF methodology was chosen given its conceptual and practical simplicity. For instance, consecutive windows do not need to overlap, as in umbrella sampling or metadynamics, with outstanding savings in simulation time.^56^ In essence, ABF computes the mean thermodynamic force along the collective variable, which is then canceled out by an equal and opposite biasing force. The inclusion of this biasing force allows the system to move seamlessly along the colvar as if the process was governed by diffusion, overcoming the metastable regions and recovering ergodicity. Theoretically, ξ is a continuous variable, but in practice the collective variable is divided into small bins of width *δξ*, where samples are accrued into each bin to compute the mean force in the interval . In all of our simulations, the bin width *δξ* is 0.5 Å, which permits to capture the fine details of the continuous PMF without affecting the number of force samples accumulated in each bin.^88^

As the simulation progresses, more samples are accrued within each bin and so the estimation of the mean force from the instantaneous thermodynamic forces *F* gets refined toward its actual value. The thermodynamic integration formalism, on which ABF is based, allows to yield the PMF as the mean force along the collective variable is simply the negative gradient of the free energy:
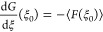
1where the angle brackets denote ensemble averages. The mean value for the force in each bin coming from these samples is used to compute the gradient as in eq 1 and, through numerical integration, the free energy value *G*. The PMF is eventually shifted by subtracting the Jacobian term, so that the free energy value at large distances is zeroed where the deposit-DCA binding interactions fade away.^87^ However, it is important to note that albeit the sampling is facilitated along ξ using the ABF approach, its convergence still depends on the relaxation of the other (orthogonal) degrees of freedom. For studying adsorption processes, this would imply the many different configurations along the planes parallel (e.g., *x* – , *y* – coordinates) to the basal axis of the PAH, as a function of the *z* – projected distance to the surface.

Another important remark in ABF-MD simulations concerns the nonequilibrium effects derived from the instantaneous force term. Since it fluctuates very strongly, a low number of samples in the beginning of the simulation implies that the estimate for the mean force will be poor, and the bias (if implemented) will very likely drive the system out of equilibrium. Therefore, the biasing force is only applied to the system once a threshold of samples has been accumulated in each bin. In our simulations, the system evolves unbiased until 500 samples have been achieved in each bin, which has been found to be a solid trade-off between delayed ABF application and unwanted nonequilibrium results.^87^ From 500 up to 1000 samples, there is a linear correction factor for the ABF force from 0 to 1, while the ABF methodology is fully applied after 1000 samples are collected in each bin.

As for other importance sampling methodologies, the analysis of convergence is far from trivial.^56^ Although there are formal mathematical proofs of the ABF long-time convergence,^89^ MD simulations are not infinite and the simulation time for satisfying the ergodic hypothesis is not known on beforehand. Therefore, it is important to check for signs of poor sampling that might affect the quality of the ABF procedure and, consequently, the PMF accuracy. Sanity checks must include uniform sampling and reversible transitions along the collective variable, besides the time evolution of the PMF profile. Examples showing the convergence of the PMF are given in the Supporting Information (Figures S1, S2 and S3).

### DFT Procedure

**Figure 3:**
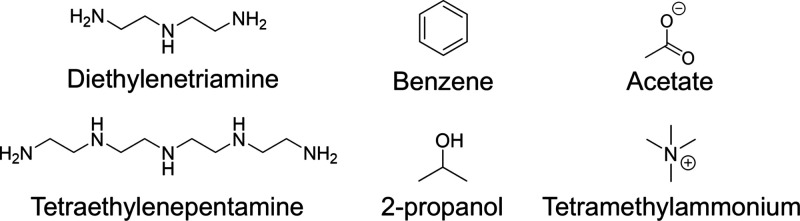
Chemical structures of the DCA fragment molecules considered in the DFT calculations, targeting the same atomistic mechanisms for binding of the detergent to PAH.

Fundamental insights into the adsorption of DCAs on carbonaceous deposits are obtained by using DFT, which help to decouple the key contributions to the adsorption process. Furthermore, this approach would permit to validate the OPLS-AA and IFF force fields used in MD simulations, as it only depends on the fundamental laws of physics and do not require any previous knowledge on the system under study. Due to the accuracy of DFT calculations, calculating the adsorption energy of the large DCA molecules would be computationally expensive and results difficult to interpret. Therefore, we instead study small molecules containing the same polar motifs as the DCAs adsorbing onto circumovalene in our MD simulations, which are presented in Figure 3.

In order to appropriately sample the conformational space, different initial geometries are modeled for all molecules from Figure 3, such as monodendate, bidendate or tridendate adsorption for TMA.^90^ Moreover, different relative locations of the adsorbate with respect to the carbonaceous deposit are studied to gauge whether adsorption is favored in the basal PAH plane or next to the circumovalene edge. DFT calculations are performed using the Vienna ab initio simulation program (VASP)^91−94^ with the projector augmented wave (PAW) formalism.^95^ Calculations are performed with the nonlocal optB86b-vdW exchange-correlation functional^96^ to approximately account for dispersion interactions, which are important to obtain accurate adsorption energies from DFT calculations of organic molecules on various surfaces.^97,98^

The plane-wave cutoff energy is set to 400 eV, with a single *k*-point in the Γ – position. The convergence criterion for the total energy in the self-consistent cycle during electronic minimization is set to 10^–6^ eV, whereas for the forces during ionic relaxation is 10^–2^ eV Å^–1^. At least 15 Å of vacuum space is kept in all of the three Cartesian coordinates so that the plane waves die out before interacting with their periodic replicas. The adsorption energy *E*_ads_, which reflects the strength and nature of the interaction between the DCA and the PAH deposit, is computed through:
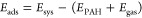
2where *E*_sys_ is the total energy of the geometrically relaxed adsorbed complex, *E*_PAH_ is the total energy of the relaxed deposit, and *E*_gas_ is the total energy of the relaxed molecular fragment in vacuum. Further insights on the nature of the deposit-DCA interactions are produced by analyzing the contributions from the individual atoms, such as the spatial resolution of the charge transfer upon adsorption using electron density difference plots. This charge redistribution is rationalized in terms of the so-called Bader charges, computed using a grid-based algorithm,^99^ that compares the location of electrons upon adsorption relative to where they are more likely to be found in the isolated molecular structures.

## Results and Discussion

### Effect of DCA and Fuel Type

**Figure 4:**
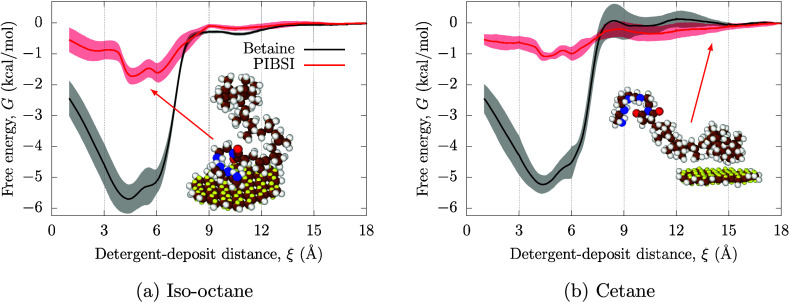
Adsorption PMFs for the PIBSI and the zwitterionic surfactant on circumovalene from (a) iso-octane and (b) *n*-hexadecane. Solid lines correspond to the mean of three independent runs, and the shaded areas represent the standard deviations, which constitute an estimate of the MD statistical error. The maximum values of these standard deviations are reported in the text. The dashed vertical lines on the *x*-axis at 3 Å intervals indicate the spatial binning for the independent ABF-MD simulations.

The PMFs for surfactant adsorption on circumovalene from pure iso-octane (gasoline) and *n*-hexadecane (diesel) are shown in Figure 4a and Figure 4b, respectively. Both base fluids share the same nonpolar chemistry and so the choice of iso-octane or *n*-hexadecane is not expected to have a large effect on the adsorption of either DCA type. On the other hand, there are major differences in the polar head-groups of the PIBSI and zwitterionic surfactants, which are expected to give significant differences in the adsorption PMFs. The nonpolar DCA tail-groups are not expected to play a significant role in adsorption because they will only have relatively weak CH – π interactions^100^ with the deposit, which will be of similar strength to those from the fuel molecules.

From Figure 4a and Figure 4b, it is clear that the adsorption of the zwitterionic surfactant onto circumovalene is much stronger than for PIBSI for both fuel surrogates. In all cases, the PMF shows a negative minimum, indicating that adsorption is exergonic due to interactions between the DCA head-groups and the PAH molecules. For PIBSI, the adsorption free energy on circumovalene is approximately −1.8 ± 0.4 kcal mol^–1^ in iso-octane and −1.0 ± 0.3 kcal mol^–1^ in *n*-hexadecane, whereas for the zwitterionic surfactant is approximately −5.8 ± 0.7 kcal mol^–1^ and −5.1 ± 0.7 kcal mol^–1^, respectively. These result are consistent with previous experimental measurements that showed enhanced adsorption strength for molecules with additional functional groups that can interact with the deposit through multiple noncovalent interactions besides the standard polyamine groups in PIBSI.^37^ The adsorption energies are comparable to previous MD studies of the adsorption of small molecules on graphene from water (−4 kcal mol^–1^ to −8 kcal mol^–1^).^57^ The somewhat weaker adsorption in our MD simulations can be attributed to the stronger interaction of the fuel solvent with the aromatic surface compared to water, the use of finite PAHs rather than infinite graphene sheets, and penalising entropic effects due to the many adsorption conformations of the DCA head-groups.

In PIBSI, the deposit-DCA interaction comes primarily from noncovalent NH – π interactions^68^ between the polyamine groups and the aromatic deposit. On the other hand, the larger number of polar moieties in the betaine headgroup underpins the improved performance of the zwitterionic surfactant. The presence of the alcohol group allows to leverage similar noncovalent forces as in the case of PIBSI, based on OH – π interactions.^68^ The phenyl group in the zwitterionic surfactant also has π–π interactions with the circumovalene deposit.^101^ Most importantly, the quarternary ammonium cation enables cation-π interactions,^67^ while the carboxylate anion results in ion-dipole or anion-π interactions.^102^

The standard OPLS-AA parametrization^71−73^ underestimates the strength of cation-π interactions, which leads to an adsorption free energy for the zwitterionic surfactant on circumovalene from iso-octane that is around one-third lower than when the π-electrons are explicitly represented using the IFF framework.^76,77^ This is similar to the underestimation in the adsorption energy for the tetramethylammonium (TMA) cation-benzene system reported previously with OPLS-AA.^75^ On the other hand, the adsorption free energy for PIBSI is identical for the standard OPLS-AA parametrization and IFF with explicit π-electrons. The PMF profiles for the standard OPLS-AA are shown in the Supporting Information (Figure S4).

**Figure 5:**
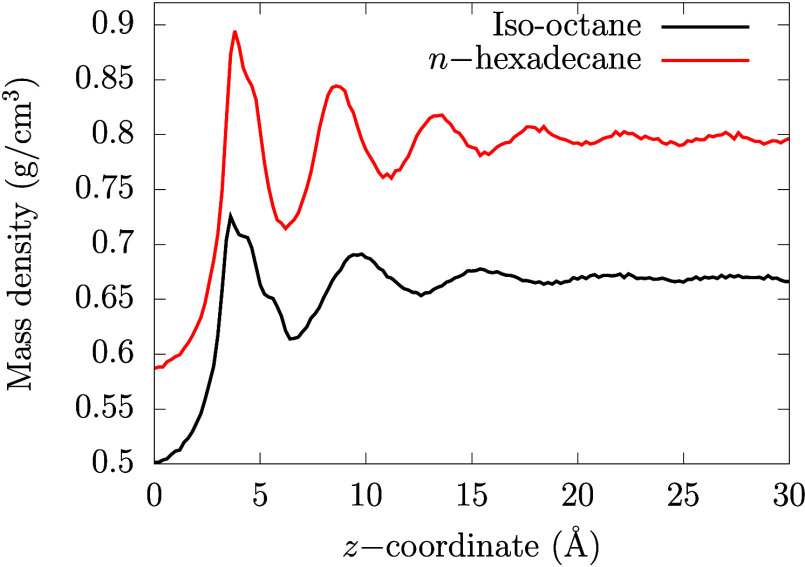
Mass density profiles for iso-octane and *n*-hexadecane in the *z*-coordinate, perpendicular to the PAH surface plane. The *z* = 0 coordinate in the *x*-axis corresponds to the PAH center of mass, i.e., the same abscissa as ξ = 0 in the PMF plots.

Comparing Figure 4a to Figure 4b shows that the adsorption is slightly weaker from *n*-hexadecane than from iso-octane. The oscillatory mass density profiles in Figure 5 show stronger layering on circumovalene for the long linear alkane *n*-hexadecane than the shorter branched alkane iso-octane. This observation is in agreement with previous MD simulations of linear and branched alkanes.^103^ The density oscillations in Figure 5 extends further into the bulk for *n*-hexadecane (∼30 Å) compared to iso-octane (∼20 Å). Moreover, the peaks in the density profile are more pronounced with respect to the bulk baseline as chain-like molecules tend to align parallel to the deposit. This stronger layering provides additional barriers to adsorption,^47^ since binding onto the surface requires the surfactant molecules to penetrate through these layers and partially remove alkane molecules.

In Figure 4, the most stable adsorption structure in the PMF occurs at similar headgroup-PAH distances (between 3 and 6 Å) for both surfactants. These distances are in good agreement with previous MD simulations of small molecule adsorption on graphene from water.^57^ Unlike those for small molecules,^57^ the PMFs for the larger surfactants of this study do not show a single sharp basin, but have the broad main feature at ∼4 Å and a less apparent shoulder at ∼6 Å. The position of the main basin corresponds to that of the first solvent mass density maximum in Figure 5, suggesting that the surfactants need to penetrate the final strongly layered solvent layer to maximize the adsorption strength. The shoulder basing of the PMF corresponds to the first density minimum, suggesting that surfactant adsorption is only slightly weaker (∼1 kcal mol^–1^) when the surfactant lies above the final solvent layer.

In Figure 4b, the PMF for both surfactants in *n*-hexadecane becomes slightly positive (endergonic) at distances of 8 Å and 12 Å, which corresponds to the second and third density maxima in Figure 5. This implies an energy barrier to adsorption due to penetration of the strongly layered final *n*-hexadecane layer, which may affect the adsorption kinetics.^47^ On the other hand, the PMF remains negative at all distance for the surfactants in iso-octane, suggesting that there is no energy barrier for adsorption even when the deposit-DCA interactions are mediated by the tail-group of the former.

### Effect of PAH Size

**Figure 6:**
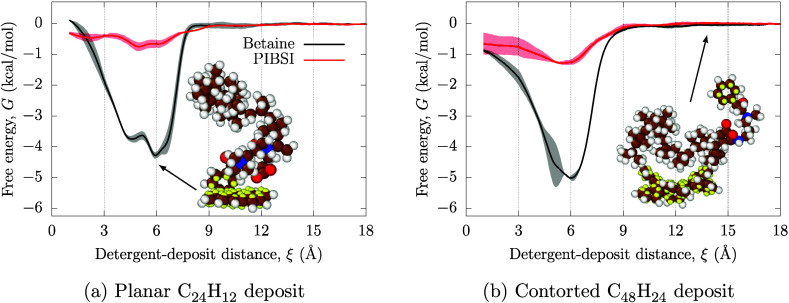
Adsorption PMFs for the PIBSI and the zwitterionic surfactants from iso-octane on (a) coronene and (b) hexa-cata-benzocoronene. Solid lines correspond to the mean of three independent runs, and the shaded areas represent the standard deviations, whose maximum values are reported in the text.

Besides circumovalene, various intermediate PAH sizes have been identified inside injector deposits.^10^ To rationalize the effect of PAH size on adsorption, we carry out a subset of simulations with the smaller PAHs using iso-octane as the base solvent, whose molecular structures are shown in Figure 1. The adsorption PMFs are shown in Figure 6a for the planar coronene (C_24_H_12_) and Figure 6b for contorted hexa-cata-benzocoronene (C_48_H_24_).

The adsorption strength decreases with decreasing deposit size, which is due to the reduced number of potential binding sites for the DCA. For the smaller coronene deposit, the PIBSI PMF becomes almost flat, with only a shallow energy minimum of −0.75 ± 0.15 kcal mol^–1^, as shown in Figure 6a. Adsorption is somewhat stronger for hexa-cata-benzocoronene with an energy minimum of −1.3 ± 0.4 kcal mol^–1^ for PIBSI, as shown in Figure 6b. The reason for these very small values is due to the PIBSI headgroup being larger than the deposit and not sufficiently flexible to maximize the number of noncovalent interactions with the PAH edge when wrapping around it. The energy minima for the smaller PAHs are at larger distances than for circumovalene, with the minima in the free energy profiles at ξ ≈ 6 Å. This suggests that the optimal adsorption conformation occurs when the DCA headgroup lies directly on top of the deposit, and that the conformations with the DCA wrapped around the deposit are not as favorable. A similar trend is found for the zwitterionic surfactant, but with much stronger adsorption. The free energy of adsorption is slightly stronger when interacting with C_48_H_24_ (−5.0 ± 0.8 kcal mol^–1^) than with C_24_H_12_ (−4.3 ± 0.5 kcal mol^–1^), highlighting once again the role played by the number of deposit binding sites.

### Contributions of Enthalpy and Entropy

To further explore the thermodynamics of the adsorption process, the PMF is decomposed into its enthalpic and entropic terms.^65^ Although strictly we are computing the Helmholtz free energy in the NVT ensemble used in the production runs, it can be assumed equivalent to the Gibbs free energy if one neglects the pressure–volume contribution.^62^ According to classical thermodynamics, the entropic term can be calculated from the temperature dependence of the free energy profile, assuming that enthalpy and entropy do not change over a small temperature range, giving:
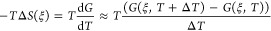
3

**Figure 7:**
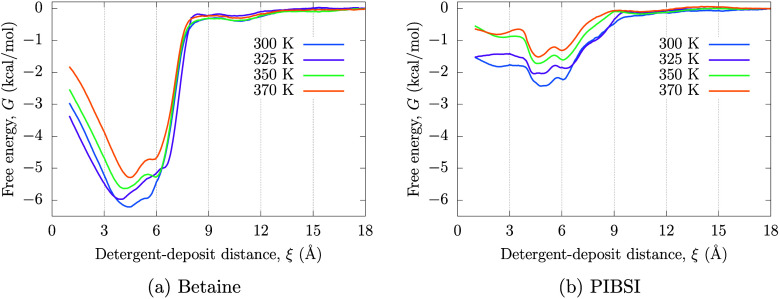
PMF at different temperatures for surfactants on circumovalene from iso-octane for (a) zwitterionic surfactant and (b) PIBSI. As characteristic of nonpolar solvents, the adsorption free energy is stronger at lower temperatures.

To extract the fundamental contributions to the adsorption free energy, simulations were carried out at different temperatures, with results being presented in Figure 7.

**Figure 8:**
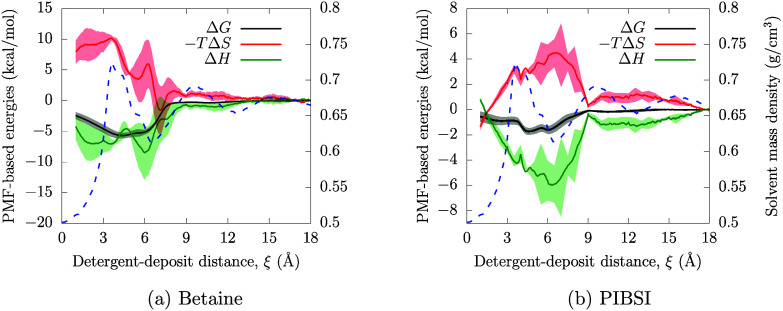
Entropic and enthalpic contributions to the PMF on circumovalene from iso-octane for (a) a zwitterionic surfactant and (b) PIBSI. The iso-octane mass density profile is also shown as a dashed blue line. The Gibbs free energy profile (black solid line) is produced at 350 K, where Δ*T* = 25 for its use in eq 3 to infer the entropic contribution.

Figure 7 shows that adsorption becomes weaker with increasing temperature for both surfactants. When water (or other polar species^46^) is used as a solvent, higher temperatures typically lead to stronger adsorption.^104^ Higher temperatures imply a larger molecular motion, because of higher kinetic energies, and so a broader range of accessible configurational states from a statistical mechanics standpoint. For the same DCA headgroup interacting with the deposit, the loss of conformational freedom upon binding is more prominent at large *T* values, from where the decrease in adsorption free energy can be attributed to entropy losses. The PMFs at different temperatures are used to derive the spatially resolved entropy (eq 3) and enthalpy contributions, presented in Figure 8. Here, the density profile for iso-octane is also included using dashed lines to illustrate the contribution of the solvent layering on the thermodynamics of the adsorption process.

From Figure 8, it is clear that the movement of the DCA from the bulk phase (large ξ values) to the PAH surface (lower ξ values) during adsorption is governed by enthalpy. The enthalpy is negative (favorable) for both surfactants at all deposit-DCA distances. For the zwitterionic surfactant, there are two clear enthalpy minima (−15 kcal mol^–1^) before and after the first solvent density peak (∼4 Å). For PIBSI, there is just one broad enthalpy minima (−6 kcal mol^–1^) above the first density peak of the solvent.

In entropy terms, there is an unfavorable increase as the DCA molecules move from the bulk to the circumovalene surface. Entropy losses are known to be significant for the adsorption of flexible molecules in a vacuum.^105^ This is because a proportion of the degrees of freedom in the DCA molecules become fixed when attaching to a deposit binding site. As such, the entropy loss is more significant for the bulkier zwitterionic headgroup than for PIBSI.^62^ When water is used as a solvent, the entropy gain by releasing adsorbed water molecules into the bulk can compensate for the loss of entropy in the surfactant molecule to give an overall favorable increase in entropy upon adsorption.^104^ In Figure 8, there is no such compensation from the iso-octane solvent since the entropy gain by releasing the solvent molecules into the bulk is less than the entropy loss of the surfactant during adsorption.

A final remark worth of discussion from Figure 8 can be seen in the enthalpy–entropy breakdown for PIBSI, where there is a transition from an entropy penalty to an entropy-driven adsorption for small distances (ξ ≲1.5 Å). This occurs because the surfactant, when expelling solvent molecules to approach the deposit, gains configurational freedom when binding onto the PAH aromatic groups, in comparison to when it is restricted to interact with adsorbed solvent. Furthermore, the enthalpic term becomes unfavorable at these small colvar values, which corresponds to the PIBSI headgroup wrapping around the edge of the PAH molecule — see the adsorption energy of PIBSI-like head-groups at the PAH edge on Table 1. This would mean that, within this range of conformations, the enthalpic contribution of PIBSI (based on the force field interactions) with PAH is less preferred in comparison to the solvent adsorption.

**Table 1:** DFT Adsorption Energies at the Top of the Deposit and around the Edge for the Base Solvent and the Different Molecular Fragments in the DCAs^a^

moiety (solvent/DCA)	*E*_ads_ at the PAH top	*E*_ads_ at the PAH edge
iso-octane (solvent)	–16.1	–1.1
DETA polyamine (PIB)	–17.3	–11.1
TEPA polyamine (PIB)	–23.5	–11.6
2-propanol (betaine)	–8.8	–3.9
benzene (betaine)	–13.0	–4.3
acetate (betaine)	–23.9	–23.7
tetramethylammonium (betaine)	–49.9	–18.9

a Energies expressed in kcal mol^–1^.

### DFT Calculations

The PMF results obtained in the ABF-MD simulations are strongly dependent on the accuracy of the force field describing the interactions between the atoms.^106^ To validate the use of OPLS-AA with the explicit modeling of π – electrons through the IFF, we carry out first-principles DFT calculations. As seen in the previous section, the base solvent plays only a minor role in the DCA adsorption process onto a PAH deposit. Therefore, the qualitative trends found in classical MD simulations with explicit modeling of the solvent can be compared with DFT results in vacuum. In particular, the adsorption enthalpy values from the MD simulations shown in Figure 8 provide the most direct comparison to the DFT calculations. Simulations are also performed for iso-octane, which permits to understand how favorable is the binding of a surfactant polar group in comparison to the solvent tendency to adsorb on circumovalene.

Considering the finite size of the circumovalene molecule, calculations are performed with different initial configurations, either on top of the deposit or around its edge, as the chemical environment is substantially different in both sites. This permits to infer the preferential binding site for each motif, depending on the governing mechanisms. The adsorption energies are presented in Table 1 for the functional groups in both DCA head-groups and for the solvent. A comparison with calculations carried out with the standard PBE functional,^107^ where the van der Waals interactions are not explicitly captured, are shown in the Supporting Information (Table S1). The adsorption energy for iso-octane (−16.1 kcal mol^–1^) on top of circumovalene in Table 1 is in reasonable agreement with previous DFT calculations for *n*-octane on graphene (−15.6 kcal mol^–1^) with a similar functional.^108^ Adsorption for iso-octane is mainly governed by van der Waals-based CH – π^100^ interactions. It must be mentioned that the absolute values of the adsorption energy depend on the functional of choice and are not supposed to be a high-fidelity interpretation of experimental adsorption enthalpies. This is why we are interested in the relative energies instead, which would permit to analyze which surfactant motifs are more prone to interact with the deposit given that the solvent is also free to bind with circumovalene.

The groups with the strongest binding in Table 1 are the cation (TMA) and anion (acetate) belonging to the zwitterionic surfactant. All the polar motifs prefer to adsorb on top of the deposit rather than wrapping around the edge, except the carboxylate anion which does not show a preferential binding site — the *E*_ads_ difference between both sites is lower than the thermal energy (∼0.7 kcal mol^–1^) at 350 K. The phenyl and the alcohol groups in the zwitterionic surfactant are not favored with respect to the base solvent, and so their contributions are left out in the following discussion.

**Figure 9:**
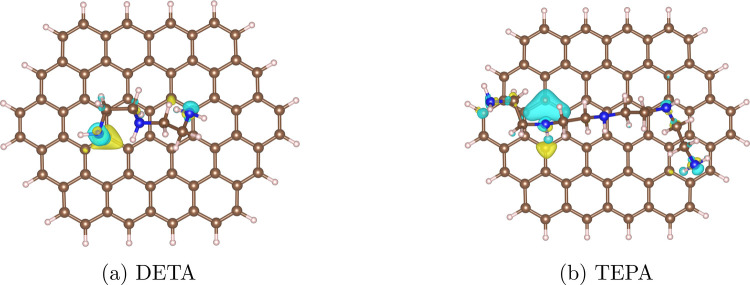
Electron density difference plots for the most stable adsorption conformations of (a) DETA and (b) TEPA on circumovalene. Yellow represents regions of charge density accumulation, whereas blue denotes charge density depletion. The isosurface level is 0.0005 e/a_0_^3^, where e represents the charge of an electron and *a*_0_ is the Bohr radius. C atoms are colored brown, H atoms white, and N atoms blue. Rendered using VESTA.^111^

Regarding the DCA based on the conventional PIBSI chemistry, it is found that the adsorption strength increases with the length of the polyamine chain, i.e. the TEPA interactions are stronger than the DETA ones, see Figure 3 for a comparison, which roughly exceed the iso-octane tendency to adsorb. Our DFT results align with calculations carried out at higher level of theory with a more accurate treatment of the correlation effects behind the noncovalent amine-π mechanisms.^109^ Although previous calculations have suggested that the adsorption is not favorable for ammonia,^110^ the dispersion effects of alkyl-substituted compounds (namely, amines) exceed the repulsive electrostatics and the overall interaction becomes attractive. Likewise, the unfavorable polyamine (either DETA or TEPA) adsorption around the edge, relative to iso-octane, agrees with the positive enthalpy values found for small ξ values in Figure 8b. To better understand the adsorption energies from Table 1, we compute the electron density difference plots (shown in Figure 9) and the Bader charges corresponding to each polyamine chain. The results show that there is no charge transfer between the adsorbate and the deposit, which is characteristic of van der Waals-based physisorption, underpinning the very small PIBSI free energies found in MD simulations, see Figure 4.

The broadest landscape of interactions is brought by the carboxylate anion, represented in DFT simulations through the acetate group. Dispersion forces do not play a major role in this case, as confirmed by DFT simulations with the methanoate ion in which the adsorption energy from eq 2 is equivalent to acetate for the basal and edge binding sites. It is the only DCA motif which does not have a preferential binding site, which showcases the larger versatility of zwitterions as DCAs in comparison to quaternary ammonium salts.^36^ Given the flexibility of the headgroup and the many degrees of freedom, the carboxylate group can interact with either the top or the edge of the deposit and, still, contribute equally to the adsorption process, which does not happen for the other motifs. Indeed, according to DFT, its strength is as large as the TEPA chain, on which the PIBSI chemistry for detergency (although targeting different deposit precursors, e.g. more acidic species) has been based for decades. However, the underpinning mechanisms are different for the carboyxlate group when the adsorption takes place around the edge or on top of the deposit. Concerning the edge binding, it is a case of ion-dipole interactions governed by classical electrostatics, where the negative charge of the carboxylate group is oriented to maximize the interaction with the positive pole of the PAH structure. When adsorption occurs on top, it consists of a somewhat counterintuitive attraction between an anion and the π–electron rich region of space, through noncovalent anion-π interactions.^112^

**Figure 10:**
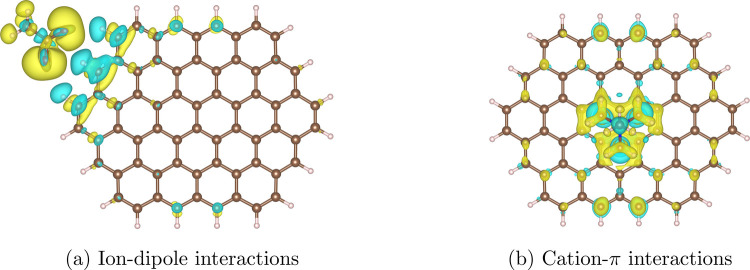
Electron density difference plots for the more stable adsorption configurations for (a) the acetate anion and (b) the TMA cation. The isosurface level is 0.001 e/a_0_^3^. C atoms are colored brown, H atoms white, O atoms red, and N atoms blue. Rendered using VESTA.^111^

The electron density difference plot for the edge adsorption of acetate on circumovalene is presented in Figure 10a. The basal site adsorption is shown in the Supporting Information (Figure S6b), with an ensuing discussion on the directionality of the charge transfer of anion-π interactions. The acetate ion shows more prominent regions of charge accumulation and depletion between adsorbate and deposit relative to Figure 9, with Bader charges transfer of 0.50 and 0.53 e in the basal and edge adsorption scenarios, respectively. The mild charge transfer from the deposit to the adsorbate is responsible for the adsorption energy value from Table 1, similar to the dispersion-dominated TEPA polyamine chain, despite being a much smaller motif.

The cation-π interactions are addressed via the TMA cation, which mimics the quaternary ammonium in the zwitterionic DCA used in MD simulations. In TMA, N carries a partial negative charge, whereas the positive charge is mostly attributed to the H atoms in the methyl groups.^67^ We carry out simulations considering the monodendate (i.e., one methyl group pointing toward the deposit), bidendate, and tridendate adsorption structures. The electron density difference plot for the most stable basal adsorption of TMA on circumovalene is shown in Figure 10b. Other conformations are shown in the Supporting Information (Figure S5). As observed elsewhere in the literature using different levels of theory,^90,113^ the adsorption energies correlate with the number of methyl groups directly interacting with the deposit, with *E*_ads_ values of −42.4 kcal mol^–1^, – 46.7 kcal mol^–1^, and −49.9 kcal mol^–1^ for the monodendate, bidendate, and tridendate structures, respectively. Similar Bader charge transfer of about −0.93 e (the charge is transferred from the adsorbate onto the deposit) are found for the three geometries, from where one can deduce that dispersion forces contribute to further stabilizing the tridendate conformation. This stronger Bader charge transfer is illustrated in Figure 10b, from which the regions of electronic accumulation and depletion are larger than in other electron density difference plots. The bottom line from TMA results is that, regardless of the geometric conformation captured in time-dependent MD simulations, the binding energy from the quaternary cation will very likely be the most relevant one for the PMF. This might help to explain why, in turn, the zwitterionic DCA is associated with a much stronger PMF in Figure 4, as the key functional groups (namely, cation and anion) are going to strongly interact with the deposit, regardless of the adsorptive configuration.

Assuming each PIBSI molecule displaces one iso-octane molecule, the energy change based on the DFT calculations of TEPA and iso-octane adsorption will be −7.4 kcal mol^–1^, which agrees well with the adsorption enthalpy from the MD simulations (−6.0 kcal mol^–1^) in Figure 8b. For the zwitterionic surfactant, assuming that two iso-octane molecules are displaced due to the larger headgroup size, the energy change based on the DFT calculations of TMA and iso-octane adsorption will be −17.7 kcal mol^–1^, which is in also reasonable agreement with the adsorption enthalpy from the MD simulations (−16.0 kcal mol^–1^), see Figure 8a. Therefore, the DFT calculations are in solid quantitative agreement with the MD simulations for the deposit-DCA systems under study.

## Conclusions

We have performed MD and DFT calculations to shed light onto the molecular mechanisms controlling the adsorption of DCAs onto model carbonaceous deposits. The MD simulations show that zwitterionic surfactants give stronger adsorption onto PAH-based deposits than conventional PIBSI from gasoline (iso-octane) and diesel (cetane) surrogates. This is due to the increased number of polar functional groups in the headgroup, which leads to stronger bonding from additional noncovalent interactions, most notably the cation-π interactions from the quaternary ammonium group. Given the physical nature of cation-π interactions, these are not captured with nonpolarizable force field parametrizations and more involved approaches are required, such as the explicit representation of π-electrons using IFF. DFT simulations, with a nonlocal functional that approximates van der Waals interactions, are used to provide insights on the fundamental mechanisms underpinning the thermodynamics of adsorption, as well as validating the energetics modeled by the force field in MD simulations. The adsorption of both DCAs is slightly stronger from iso-octane than from cetane, which is due to the more pronounced molecular layering of the latter on the PAH surface. The DCAs also adsorb stronger to larger PAHs because this enables them to bind through a larger number of functional groups. We decompose the adsorption free energies from the MD simulations into entropic and enthalpic components, and find that the enthalpy term dominates for both surfactants. This observation contrasts to surfactant adsorption from aqueous solution, where there is usually an increase in entropy due to the release of surface-bound water molecules.

Our proposed multiscale modeling methodology can be extended to study adsorption at solid–liquid interfaces for a wide range of applications. Pertinent examples include other important factors affecting deposit formation in ICEs, such as interactions with biocomponents (e.g., ethanol),^46^ the interplay with other additives (e.g., friction modifiers),^114^ and the effect of common contaminants (e.g., water).^115^ Since real ICE deposits are more complex than the simple PAHs considered here, future studies could also investigate different deposit chemistries, such as carbonaceous deposits containing oxygen,^17^ nitrogen^116^ or metal salts.^4^ Likewise, the methods developed can also be readily extended to investigate surfactant adsorption on PAHs for other applications such as environmental remediation^117^ and dispersant additives in lubricants.^114^

## Supporting Information Available

Colvars configuration file, PMF convergence studies, free energy profiles using OPLS-AA without π-electrons, and further insights from the DFT calculations (PDF)
