# Supplementary material for: Molecular Insights into the Adsorption of Deposit Control Additives from Hydrocarbon Fuels
Source: Langmuir. 2025 Jan 16;41(3):1900–13. doi: 10.1021/acs.langmuir.4c04368 (PMC11780739; doi:10.1021/acs.langmuir.4c04368)
Supplement: Supplementary file 1 — la4c04368_si_001.pdf [file la4c04368_si_001.pdf]

Supporting Information for: Molecular Insights into the Adsorption of Deposit Control Additives from Hydrocarbon Fuels

Carlos Corral-Casas,*,† Carlos Ayestarán Latorre,† Chiara Gattinoni,‡ Mark Brewer,¶ Jörn Karl,§ Daniele Dini,† and James P. Ewen*,†

†*Department of Mechanical Engineering, Imperial College London, South Kensington Campus, London SW7 2BX, United Kingdom*

‡*Department of Physics, King's College London, Strand Campus, London WC2R 2LS, United Kingdom*

¶*Shell Global Solutions International B.V., Grasweg 39, 1031 HW Amsterdam, Netherlands*

§*Shell Global Solutions (Deutschland) GmbH, Hohe-Schaar-Straße 36, 21107 Hamburg, Germany*

E-mail: c.corral-casas@imperial.ac.uk; j.ewen@imperial.ac.uk

Contents

1 Colvars configuration file for an ABF-MD simulation **S2**

2 Convergence of the PMF **S3**

3 PMF using OPLS-AA without $\pi-$electrons **S5**

4 Complementary discussions on DFT calculations **S8**

1 Colvars configuration file for an ABF-MD simulation

The colvars[1] configuration file is a script that defines collective variables, general parameters, and biases for carrying out MD simulations with enhanced sampling. To enable a colvars-based calculation in LAMMPS,[2] one can set a `fix` command where the only required argument is the name of the colvars configuration file:

```
fix ID all colvars *configfile* keyword value ...,
```

taking into account that an ABF simulation should not be run without a thermostat.

A complete configuration file to run a MD-ABF simulation in which the collective variable is the z-projected distance between the centers-of-mass of the deposit and the detergent head-group, while restraining the colvar to a region of phase space (i.e. stratification), would read:

```
## Global keywords ##
colvarsTrajFrequency 100000
indexFile detergent.ndx
indexFile deposit_free.ndx
## Colvar definition ##
colvar {
  name dist2surf
  width 0.05
  lowerBoundary 0
  upperBoundary 3
  distanceZ {
    main indexGroup  detergent
```

```
    ref  indexGroup  deposit_free }  }
## Harmonic wall for stratification ##
harmonicWalls {
  name walls
  colvars dist2surf
  lowerWalls 0
  upperWalls 3
  lowerWallConstant 80.0
  upperWallConstant 80.0 }
## ABF importance sampling ##
abf {
  name ABF
  colvars dist2surf
  fullSamples 1000
  historyFreq 1000000 }
```

2 Convergence of the PMF

The ABF methodology is based on the computation of the mean force (from the force field terms) along the collective variable in an unconstrained manner, which is afterwards cancelled out by an equal and opposite biasing force.[3] In this way, the system can erase the barriers from the potential energy surface and move along the phase space as if the dynamics of the system were dictated by a self-diffusion process. Importantly, a flat free energy landscape implies a uniform sampling along ξ, by which it becomes feasible to retrieve a representative PMF in a simulation of a realistic time scale.

To analyse whether numerical convergence of the ABF method has been obtained or not, we first look at the time evolution of the histogram of collected samples. Theoretically, the

ratio of the histogram maximum over its minimum should decrease with time and converge to 1 for very long sampling. The samples in each bin should increase uniformly and the histogram should not present new features once convergence is achieved, as presented in Figure S1a. In this range of the collective variable, the ratio of the histogram maximum over

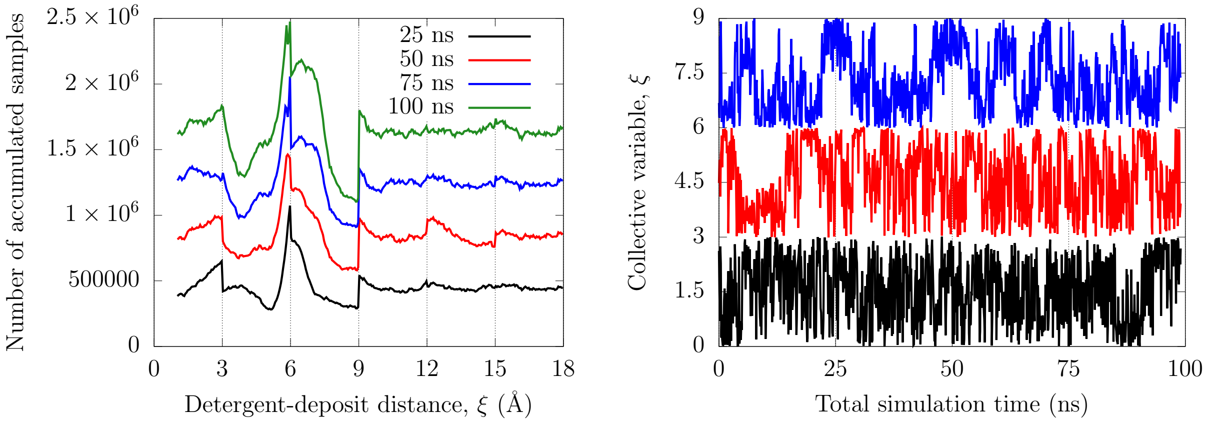

(a) Histogram of samples

(b) Colvar dynamics

Figure S1: (a) Sampling along the collective variable in different windows. Ideally, no bin should be visited less than half of the average sampling. (b) Reversibility of the transformation derived from the ABF algorithm. Black line is the trajectory of ξ in the first simulation window (up to 3 Å), red line for the second simulation window (between 3 Å and 6 Å), and blue line denotes the third simulation window (between 6 Å and 9 Å).

its minimum decreases with increasing simulation time (from 3.8 at 25 ns to 2.2 at 100 ns). The number of samples in this range varies from 1 100 000 to 2 500 000, which corrresponds to variations of the biased free energy not exceeding 0.5 kcal mol^{-1}.[3] Another straightforward indication of fine sampling is tracking the dynamics of the collective variable, presented in Figure S1b. By plotting ξ as a function of time, it can be easily observed how reversible the transition is, i.e. whether the system tends to spend a fair amount of time on a metastable state or, conversely, is able to quickly transition between states (ideal scenario).

Nevertheless, the more natural criterion for analysing the convergence relies on evaluating the evolution of the PMF with time. The free energy profiles at different simulation times are presented in Figure S2, from where it can be argued that convergence has been already achieved for simulation times of 50 ns, as for lower times the only feature that is not captured

is the shoulder at $\xi = 6$ Å.

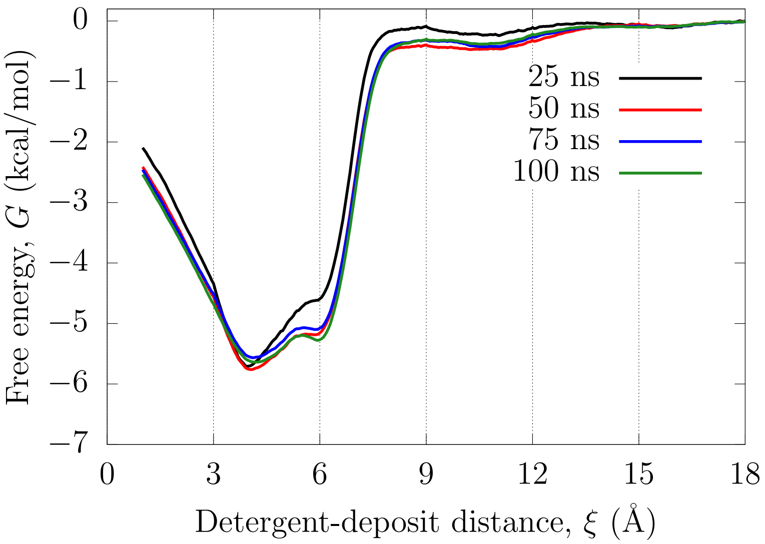

Figure S2: Time evolution of the potential of mean force for the zwitterion adsorption on circumovalene from iso-octane. The features on the free energy profiles hint that convergence has been found for 50 ns.

In Figure S3, we also extend the range of the transition coordinate, to show that cutting it off at a $z-$projected distance of zero is not hampering the ergodicity of the system. Potentially, this issue might be more relevant in a non-symmetric structure such as hexa-cata-benzocoronene, where the adsorption energy might change when adsorbing from above or below the deposit if the ergodic condition is not satisfied. It can be observed that the PMF is symmetric around $\xi = 0$ in the $[-6, 6]$ range, and therefore there are no differences in adsorption free energy due to the relative orientation of the detergent. In other words, the stratification strategy is not affecting the appropriate inspection of the complex conformational space.

3 PMF using OPLS-AA without $\pi-$electrons

The Coulombic interaction between the anion and the edges of circumovalene is readily captured by additive force fields, where partial atomic charges are used to approximate

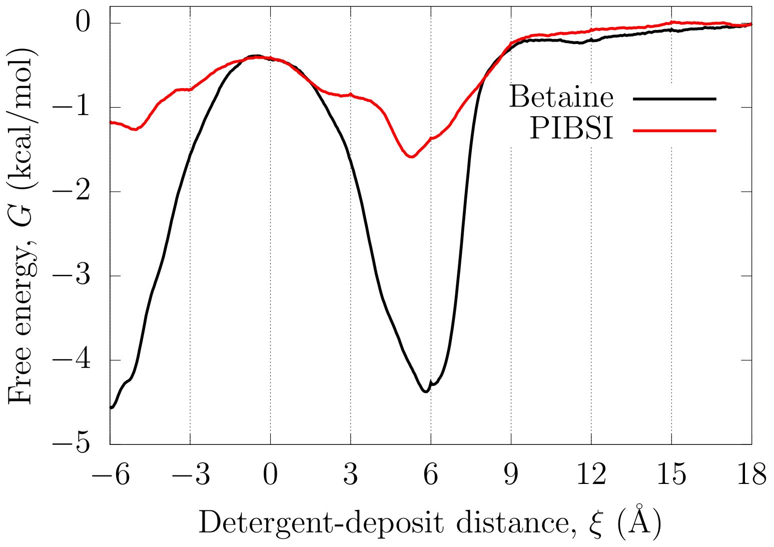

Figure S3: Cutting off the collective variable range at an arbitrary value of $\xi = 0$ is not affecting the ABF capabilities to study all of the necessary binding conformations. The PMFs correspond to a simulation time of 100 ns from iso-octane.

the distribution of electron densities within a molecule. However, the classical modelling of cation$-\pi$ interactions is not straightforward and the strength of these interactions is usually underestimated. Given the nature of ion-induced dipole interactions, the Lennard-Jones and Coulombic terms cannot capture the actual strength of cation$-\pi$ interactions.[4] These mechanisms might be rather strong, similar in magnitude to weak covalent bonds,[5] and thus its proper modelling is central to the current work for screening the detergent performance. To account for these interactions, in this work we opted for the INTERFACE-FF framework[6,7] as it is easily extended to the OPLS-AA force field without the need of further parametrisation. The addition of the $\pi-$electrons as virtual particles is carried out using a proprietary script, using 2-body and 3-body terms that reproduce the strength of cation$-\pi$ interactions measured in experimental setups.

To further understand the relevance of the cation-π interactions in the current system, we perform simulations in which the virtual particles are not included, instead using the standard OPLS-AA parameters for aromatic carbon atoms.[8–10] The PMFs are presented in Figure S4, where it can be observed that the PIBSI interactions are not affected, and its

PMF is indistinguishable from the free energy profile shown in Figure 4 in the main text where the $\pi-$electrons are included. This is not unexpected as the polyamine head-group does not contain any positively charged ion inducing a dipole on the PAH surface.

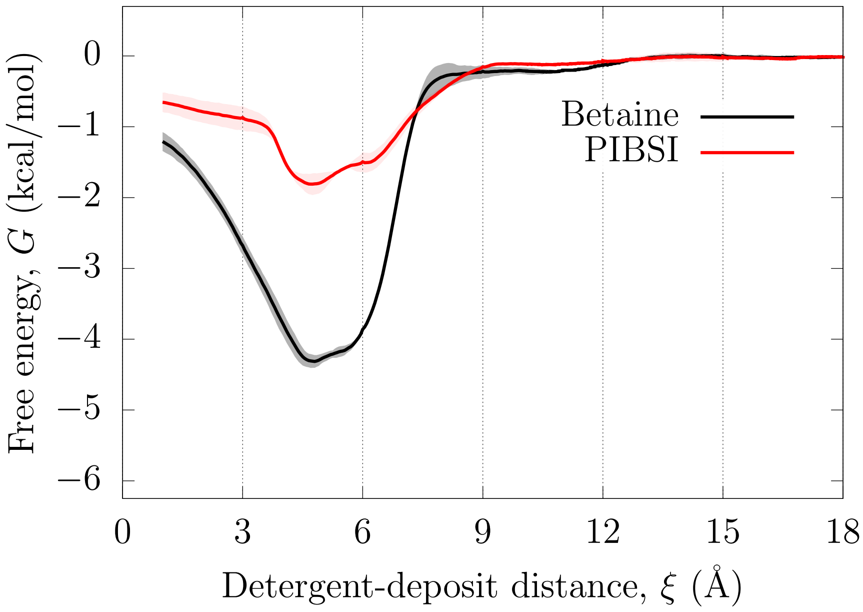

Figure S4: Adsorption PMF for the OPLS-AA parametrisation without including virtual particles representing the $\pi-$electrons. Solid lines represent the average from three independent runs, and shaded areas denote the standard deviation with respect to the mean. Base solvent is iso-octane for both detergents.

On the other hand, the binding strength for the zwitterionic surfactant comprising a cation decreases by around a third ($-4.2\,\mathrm{kcal\,mol^{-1}}$) when compared to the IFF-based model in which the $\pi-$electrons are explicitly included as virtual particles, see Figure 4. This results stresses the importance of appropriately representing the π-electrons landscape to model the adsorption energetics of cationic, anionic or zwitterionic surfactants, in particular when the absolute values of the free energies are as low as for the system under study.

4 Complementary discussions on DFT calculations

In this section, we first emphasise the need to use non-local functionals that approximate Van der Waals interactions to carry out calculations addressing non-covalent mechanisms at the DFT level of theory. These effects are not usually captured in standard implementations, such as in generalised gradient approximations of the exchange-correlation functional, which do not properly capture the long-range correlation effects between the constantly moving electrons. Here, we introduce calculations using the Perdew-Burke-Ernzerhof (PBE) functional[11] and compare them with the non-local optB86b-vdW treatment.[12] Elsewhere, it was found that a similar non-local definition of exchange and correlation effects was needed to faithfully capture the adsorption enthalpies on similar systems, consisting of small organic molecules binding on top of coronene.[13] In Table S1 we compare the use of PBE and non local-functionals for DFT energies, showing the importance of including the dispersion interactions in the exchange-correlation term.

Table S1: DFT adsorption energies, at the optB86b-vdW and PBE levels of theory, for the different molecular fragments included in the detergent head-group formulations. Binding occurs on top of the PAH. Iso-octane adsorption is also studied to compare the process energetics. Energies expressed in $\mathrm{kcal\,mol^{-1}}$

Moiety (detergent)	E_{ads} optB86b-vdW	E_{ads} PBE
Iso-octane (Solvent)	−16.1	−0.6
DETA polyamine (PIBSI)	−17.3	−5.2
2-propanol (Betaine)	−8.8	−0.4
Benzene (Betaine)	−13.0	0.3
Tetramethylammonium, $N(CH_3)_4{}^+$ (Betaine)	−49.9	−38.9
Acetate, CH_3COO^- (Betaine)	−23.9	−22.1

In those cases in which these dispersion forces are not as relevant, such as in the charged motifs, the difference between both functionals is not as importance given that other mechanisms are relatively more important, e.g. cation-π, anion-π interactions, or ion-dipole. Nevertheless, this Van der Waals term will always play a very important role in stabilisiling the complexes because of the PAH electronic structure, consisting of an electronic cloud of delocalised electrons taking part in π-bonds of the aromatic system. For instance, the electron

density difference plot for the most stable conformations of the TMA cation, undergoing tridendate binding, is shown in the main text. Slightly less stable conformations for this group would involve the monodendate (Figure S5a) and bidendedate structures (Figure S5b) with one and two methyl groups pointing down, respectively, which are shown below. However,

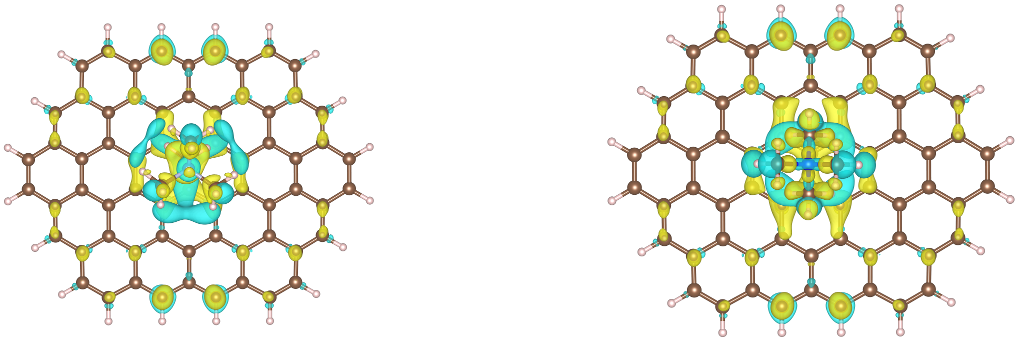

(a) Monodendate TMA adsorption (b) Bidendate TMA adsorption

Figure S5: Electron density difference plots for (a) monodendate and (b) bidendate binding of the TMA cation. The isosurface level is $0.001\,\mathrm{e/a_0^3}$.

the number of H atoms undergoing direct electrostatic interactions with the deposit for the most stable case is the same as in the monodendate structure — namely, three. This is a clear example of the further stabilisation brought by dispersion-like forces, as the largest binding strength is identified with the case in which more methyl groups are pointing down, which interact with the deposit through Van der Waals based CH-π interactions.

Another issue worth of discussion is the counter-intuitive anion-π interactions governing the adsorption of acetate on top of circumovalene. Despite the interaction is known to be repulsive for small unsubstituted aromatic systems such as benzene, the physics change with larger systems characterised by unoccupied p-orbitals such as graphene.[14] We test this principle by carrying out DFT simulations with smaller aromatic species, such as benzene and the 3-ring linear group known as anthracene, in which the adsorption energies are $E_{ads} = -6.6\,\mathrm{kcal\,mol^{-1}}$ and $E_{ads} = -18.0\,\mathrm{kcal\,mol^{-1}}$, respectively, using the aforementioned non-local functional.[12] The electron density difference plot for the adsorption of the acetate ion on top of the basal plane of anthracene is shown in Figure S6a. The Bader

charges for the adsorption on benzene and anthracene are 0.16 e and 0.42 e, in line with the increasing adsorption energies with larger aromatic rings. Given the complexity of these interactions, we also perform runs with a more involved exchange-correlation functional, such as SCAN+rvv10, which should provide a more realistic modelling of these phenomena. This approach supplements the meta-generalised gradient approximation for short-range interactions with long-range Van der Waals contributions.[15]

Still, similar results are found with respect to the optB86b-vdW level of theory, as for benzene and anthracene the adsorption energies are weak but favourable, with larger charge transfers for increasing PAH surface areas. This pinpoints the need for more accurate methods (hybrid functionals with exact exchange or post Hartree-Fock to include correlation effects) to treat exchange and correlation effects in order to grasp the fine details, as the interaction is favourable even for the smallest aromatic. For circumovalene, the adsorption is stronger ($E_{ads} = -23.9\,\mathrm{kcal\,mol^{-1}}$) but the directionality is opposite to that found for Mulliken charge analysis,[16] see Figure S6b, with charge accumulation next to the electronegative O species instead of in the carbon rings. However, this might be a case of dependence on the

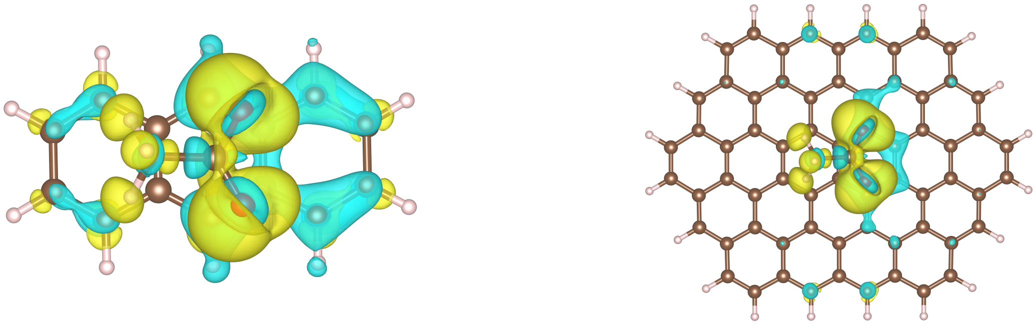

(a) Anion-π on anthracene (b) Anion-π on circumovalene

Figure S6: Electron density difference plots for basal adsorption of the acetate ion on (a) anthracene and (b) circumovalene, where the attractive interaction is governed by anion-π interactions. The isosurface level is $0.001\,\mathrm{e/a_0^3}$.

choice of methodology for density partitioning, knowing that all schemes bear some degree of arbitrariness. Indeed, different directionality in terms of the charge transfer between adsorbate and adsorbent have been found elsewhere for the adsorption of small organic molecules

on graphene when using Mulliken and Bader partitioning schemes.[17]

References

(1) Fiorin, G.; Klein, M. L.; Hénin, J. Using collective variables to drive molecular dynamics simulations. *Mol. Phys.* **2013**, *111*, 3345–3362.

(2) Thompson, A. P.; Aktulga, H. M.; Berger, R.; Bolintineanu, D. S.; Brown, W. M.; Crozier, P. S.; in 't Veld, P. J.; Kohlmeyer, A.; Moore, S. G.; Nguyen, T. D.; Shan, R.; Stevens, M. J.; Tranchida, J.; Trott, C.; Plimpton, S. J. LAMMPS - a flexible simulation tool for particle-based materials modeling at the atomic, meso, and continuum scales. *Comput. Phys. Comms.* **2022**, *271*, 108171.

(3) Comer, J.; Gumbart, J. C.; Hénin, J.; Lelievre, T.; Pohorille, A.; Chipot, C. The adaptive biasing force method: Everything you always wanted to know but were afraid to ask. *J. Phys. Chem. B* **2015**, *119*, 1129–1151.

(4) Caldwell, J. W.; Kollman, P. A. Cation-π Interactions: Nonadditive Effects Are Critical in Their Accurate Representation. *J. Am. Chem. Soc.* **1995**, *117*, 4177–4178.

(5) Dharmawardhana, C. C.; Kanhaiya, K.; Lin, T. J.; Garley, A.; Knecht, M. R.; Zhou, J.; Miao, J.; Heinz, H. Reliable computational design of biological-inorganic materials to the large nanometer scale using Interface-FF. *Mol. Simul.* **2017**, *43*, 1394–1405.

(6) Pramanik, C.; Gissinger, J. R.; Kumar, S.; Heinz, H. Carbon Nanotube Dispersion in Solvents and Polymer Solutions: Mechanisms, Assembly, and Preferences. *ACS Nano* **2017**, *11*, 12805–12816.

(7) Pramanik, C.; Jamil, T.; Gissinger, J. R.; Guittet, D.; Arias-Monje, P. J.; Kumar, S.; Heinz, H. Polyacrylonitrile Interactions with Carbon Nanotubes in Solution: Confor-

mations and Binding as a Function of Solvent, Temperature, and Concentration. *Adv. Funct. Mater.* **2019**, *29*, 1905247.

(8) Jorgensen, W. L.; Maxwell, D. S.; Tirado-Rives, J. Development and Testing of the OPLS All-Atom Force Field on Conformational Energetics and Properties of Organic Liquids. *J. Am. Chem. Soc.* **1996**, *118*, 11225–11236.

(9) Rizzo, R. C.; Jorgensen, W. L. OPLS all-atom model for amines: Resolution of the amine hydration problem. *J. Am. Chem. Soc.* **1999**, *121*, 4827–4836.

(10) Price, M. L. P.; Ostrovsky, D.; Jorgensen, W. L. Gas-phase and liquid-state properties of esters, nitriles, and nitro compounds with the OPLS-AA force field. *J. Comput. Chem.* **2001**, *22*, 1340–1352.

(11) Perdew, J. P.; Burke, K.; Ernzerhof, M. Generalized Gradient Approximation Made Simple. *Phys. Rev. Lett.* **1996**, *77*, 3865–3868.

(12) Klimeš, J.; Bowler, D. R.; Michaelides, A. van der Waals density functionals applied to solids. *Phys. Rev. B* **2011**, *83*, 195131.

(13) Lazar, P.; Karlicky, F.; Jurecka, P.; Kocman, M.; Otyepkova, E.; Safarova, K.; Otyepka, M. Adsorption of Small Organic Molecules on Graphene. *J. Am. Chem. Soc.* **2013**, *135*, 6372–6377.

(14) Shi, G.; Ding, Y.; Fang, H. Unexpectedly strong anion–π interactions on the graphene flakes. *J. Comput. Chem.* **2012**, *33*, 1328–1337.

(15) Peng, H.; Yang, Z.-H.; Perdew, J. P.; Sun, J. Versatile van der Waals Density Functional Based on a Meta-Generalized Gradient Approximation. *Phys. Rev. X* **2016**, *6*, 041005.

(16) Xiaozhen, F.; Xing, L.; Zhenglin, H.; Kaiyuan, Z.; Guosheng, S. DFT sutdy of common anions adsorption at graphene surface due to anion-π interaction. *J. Mol. Model.* **2022**, *28*, 225.

(17) Gerber, I. C.; Poteau, R. Critical assessment of charge transfer estimates in non-covalent graphene doping. *Theor. Chem. Acc.* **2018**, *137*.
